# Re: Fluoroless endourological surgery for high burden renal and proximal ureteric stones: A safe technique for experienced surgeons

**DOI:** 10.1080/2090598X.2021.1901358

**Published:** 2021-03-18

**Authors:** Ahmed R. El-Nahas

**Affiliations:** Professor of Urology, Urology and Nephrology Center, Mansoura University, Mansoura, Egypt

I have read with interest this retrospective study that reviewed the results of fluoroless endourological treatment of 183 cases of complex renal and ureteric stones. All cases were performed by an experienced single surgeon without using fluoroscopy at all [1]. Flexible ureteroscopy through a ureteric access sheath (UAS) was performed for all patients. In addition, mini-percutaneous nephrolithotomy under ureteroscopic control in the supine position was needed in 15% of patients because of a large stone burden. The stone-free rate was 92% and no patients developed Grade III or IV Clavien–Dindo complications. The authors concluded that fluoroless ureteroscopy and a mini-percutaneous approach under flexible ureteroscopic control are feasible and safe procedures in the hands of an experienced surgeon.

The rational of omitting fluoroscopy to decrease X-ray exposure for the patient and the operating room staff is valid. Some retrospective studies reported radiation-free flexible ureteroscopy [2,3]. However, there are critical steps that need the use of an imaging modality for confirmation of the instruments’ location inside the pelvicalyceal system to ensure optimal safety of the procedure. Despite the report by Aboutaleb [4] that showed the safety of fluoroless UAS insertion, it is dangerous to introduce the sheath up the ureter depending only on tactile feedback. Pulsed fluoroscopy is an alternative to conventional fluoroscopy to decrease X-ray exposure and maintain safety at the same time. Elkoushy et al. [4] reported that pulsed fluoroscopy during ureteroscopy significantly decreased X-ray exposure.

The conclusions of this manuscript must be taken with great caution because an experienced surgeon may sometimes accomplish extraordinary techniques because his high level of expertise enables him to achieve a high success rate with minimal complications. Therefore, it is unsafe for generalisation to all cases or all surgeons depending on the results of anecdotal experience [5].
